# Effects of transcranial ultrasound stimulation pulsed at 40 Hz on Aβ plaques and brain rhythms in 5×FAD mice

**DOI:** 10.1186/s40035-021-00274-x

**Published:** 2021-12-07

**Authors:** Mincheol Park, Gia Minh Hoang, Thien Nguyen, Eunkyung Lee, Hyun Jin Jung, Youngshik Choe, Moon Hwan Lee, Jae Youn Hwang, Jae Gwan Kim, Tae Kim

**Affiliations:** 1 Department of Biomedical Science and Engineering, Gwangju Institute of Science and Technology, Gwangju, 61005 Republic of Korea; 2 Korea Brain Research Institute, Daegu, 41062 Republic of Korea; 3 Department of Information and Communication Engineering, Daegu Gyeongbuk Institute of Science and Technology, Daegu, 42988 Republic of Korea

**Keywords:** Transcranial ultrasound stimulation, Gamma band oscillation, Amyloid-β plaques, Alzheimer’s disease

## Abstract

**Background:**

Alzheimer’s disease (AD) is the most common cause of dementia, and is characterized by amyloid-β (Aβ) plaques and tauopathy. Reducing Aβ has been considered a major AD treatment strategy in pharmacological and non-pharmacological approaches. Impairment of gamma oscillations, which play an important role in perception and cognitive function, has been shown in mouse AD models and human patients. Recently, the therapeutic effect of gamma entrainment in AD mouse models has been reported. Given that ultrasound is an emerging neuromodulation modality, we investigated the effect of ultrasound stimulation pulsed at gamma frequency (40 Hz) in an AD mouse model.

**Methods:**

We implanted electroencephalogram (EEG) electrodes and a piezo-ceramic disc ultrasound transducer on the skull surface of 6-month-old 5×FAD and wild-type control mice (*n* = 12 and 6, respectively). Six 5×FAD mice were treated with two-hour ultrasound stimulation at 40 Hz daily for two weeks, and the other six mice received sham treatment. Soluble and insoluble Aβ levels in the brain were measured by enzyme-linked immunosorbent assay. Spontaneous EEG gamma power was computed by wavelet analysis, and the brain connectivity was examined with phase-locking value and cross-frequency phase-amplitude coupling.

**Results:**

We found that the total Aβ42 levels, especially insoluble Aβ42, in the treatment group decreased in pre- and infra-limbic cortex (PIL) compared to that of the sham treatment group. A reduction in the number of Aβ plaques was also observed in the hippocampus. There was no increase in microbleeding in the transcranial ultrasound stimulation (tUS) group. In addition, the length and number of microglial processes decreased in PIL and hippocampus. Encelphalographic spontaneous gamma power was increased, and cross-frequency coupling was normalized, implying functional improvement after tUS stimulation.

**Conclusion:**

These results suggest that the transcranial ultrasound-based gamma-band entrainment technique can be an effective therapy for AD by reducing the Aβ load and improving brain connectivity.

**Supplementary Information:**

The online version contains supplementary material available at 10.1186/s40035-021-00274-x.

## Introduction

Alzheimer’s disease (AD) is one of the most common neurodegenerative diseases that affects over 50 million people in the world. AD is characterized by cognitive deficits, impairment of activities of daily living, and behavioral disturbances [1], and has two major pathological hallmarks, the presence of extracellular senile plaques caused by accumulation of Aβ, and the intracellular neurofibrillary tangles formed from the deposition of hyper-phosphorylated tau protein [2–4]. Under normal conditions, Aβ plaques are degraded by microglia and astrocytes [5] and soluble Aβ is removed through the perivascular pathway [6, 7]. The progressive shift from soluble to insoluble brain Aβ pools and the impaired plaque clearance are linked to the onset and progression of AD. Although medications currently fail to prevent the aggregation of amyloid plaques in AD patients [8, 9], pharmacological and non-pharmacological treatments based on the amyloid hypothesis are still major goals in AD treatment research, especially those that aim at reducing the accumulated Aβ [10].

Neuronal activity with different frequencies of gamma-band (~ 30 to 100 Hz) oscillation occurs across multiple brain regions, where it is assumed to play an important role in perception and cognitive functions such as attention, learning, and memory encoding and retrieval [11, 12]. Gamma oscillations are produced by synaptic activity between GABAergic inhibitory interneurons and excitatory pyramidal cells [13–15]. Degeneration of spontaneous gamma synchronization and reduction in spontaneous gamma power have typically been observed in multiple AD mouse models [16–19] and human patients [20–22].

Recently, a technique based on gamma-band entrainment is emerging as a therapeutic treatment for AD. Iaccarino et al. [23] have shown that optogenetic stimulation and visual stimulation at 40 Hz decreased Aβ peptides and increased microglia clustering around Aβ plaques in the hippocampus and visual cortex in 5×FAD mice, respectively. In addition, Martorell et al. [24] reported that multi-sensory gamma stimulation can reduce Aβ plaques and improve cognitive function. However, there is currently a lack of studies regarding the therapeutic effects of gamma-band entrainment by ultrasound stimulation for AD. Transcranially delivered ultrasound can safely activate the central neural circuits and exert neuroprotective effects on dementia [25–27]. Bobola et al. [28] delivered transcranial focused ultrasound stimulation with a 2.0 MHz carrier frequency at 40 Hz pulse repetition frequency into 5×FAD mice for 1 h per day to one hemisphere of the brain, and found a reduction of Aβ plaques and activation of microglia co-localized with Aβ in the treated area [28]. In this study, we set out to investigate the ultrasound-based gamma-band entrainment technique as a method to reduce brain pathology in an AD mouse model.

## Methods

### Animals

All animals were housed in a temperature- and humidity-controlled room at 20 ± 2 °C with 55% ± 5% humidity under a 12:12 light–dark cycle. We used 6-month-old male 5×FAD mice expressing human Aβ peptide precursor gene with Swedish, Florida, and London mutations and PS1 with mutations M146L and L286V, as at this age the 5×FAD mice show high Aβ burdens and memory impairment [29]. All animal procedures were approved by the ethics committee (GIST-2020–031), and fulfilled Association for Assessment and Accreditation of Laboratory Animal Care International guidelines.

### Surgical preparation

**Fig. 1:**
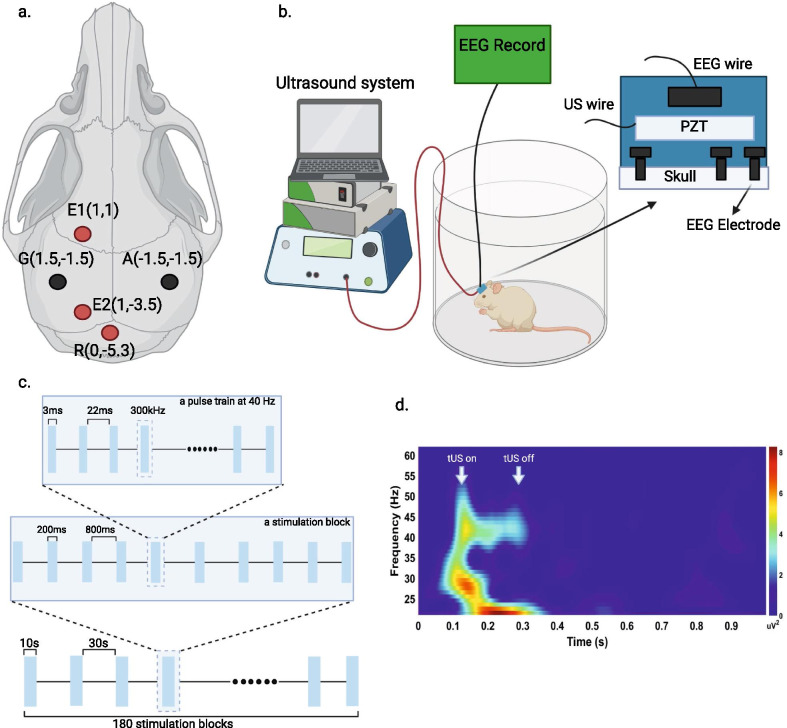
Experiment protocol. (**a**) EEG electrode positions for surgical implantation. E1 and E2 indicate electrodes on the frontal and parietal cortex, respectively, while reference electrode (R) was placed on cerebellum. G and A correspond to the electrodes for animal grounding and the anchoring screw, respectively. Red dots denote the location of implants for EEG signal generation; black dots represent implants generating no signal. (**b**) tUS and EEG recording device setup. A piezo-ceramic transducer (PZT) with two connecting wires was placed on top of the head-mount layer, which included an EEG electrode and was attached with dental cement. (**c**) Ultrasound parameter protocol. A 3-ms ultrasound pulse train at 300 kHz was followed by a 22-ms intervening period to make a carrier frequency of 40 Hz. A 10-s stimulation block followed by a 30-s resting period was repeated 180 times. (**d**) Time–frequency analysis of EEG power generated by a tUS stimulation block. A representative figure demonstrates that the EEG power was increased at 40 Hz during the tUS stimulation

Mice were anesthetized with 4% isoflurane and maintained with 0.5%–1.5% isofluorane in a stereotaxic frame. Ketoprofen (5 mg/kg) was injected subcutaneously before surgery as an analgesic. Electrodes for electroencephalogram (EEG) were implanted on the frontal (AP = 1.0 mm; ML = 1.0 mm) and parietal (AP =  − 3.5 mm; ML = 1.0 mm) areas of the skull (Fig. 1a) and connected to a premanufactured head mount (8402, Pinnacle Technology, Lawrence, KS). A piezo-ceramic disc transducer (SMD07T03R411, Steiner & Martins Inc., Davenport, FL) with two connecting wires was placed on top of the headmount layer and combined by dental cement (Fig. 1b). We gave at least one week of recovery after surgery.

### Transcranial ultrasound stimulation (tUS) experiment

Twelve male 5×FAD mice were assigned to the tUS treatment group (*n* = 6; Tg/Stim+) and the sham treatment group (*n* = 6; Tg/Stim-), and wild-type mice (*n* = 6; WT) without tUS treatment were assigned to the control group. The tUS was performed every day for two weeks. We used the piezo-ceramic disc transducer implanted to the mouse head above the EEG electrode layer. The 300 kHz ultrasonic beams pulsed at 40 Hz with 3-ms pulse width were applied transcranially. The stimulation trains of 200-ms ON and 800-ms OFF periods were repeated in stimulation blocks of 10-s ON and 30-s OFF periods (Fig. 1c). The duration of stimulation session was 2 h daily and repeated for 2 weeks. The ultrasonic beam was applied to the entire brain. The width of the ultrasound beam ranged from 5.5 to 6.0 mm. We used LabView (National Instruments Corporation, Austin, TX) to control the function generator. The signal generated by the function generator was amplified before being transferred to the piezo-ceramic disc transducer. The tUS pulsed at 40 Hz successfully induced EEG responses centered at 40 Hz, confirmed by averaging the ~ 1200 time–frequency plots for each mouse during 2 h of stimulation (Fig. 1d). We used ultrasound with a spatial peak pulse average intensity (I_SPPA_) value of 1.2 W/cm^2^ and a spatial peak time average intensity (I_SPTA_) value of 14.4 mW/cm^2^. The sonication parameters selected are considered safe and below the limits set by the US Food and Drug Administration for diagnostic ultrasound imaging (I_SPTA_ 720 mW/cm^2^, I_SPPA_190 W/cm^2^, Mechanical Index 1.9). The mice were sacrificed within 24 h after the last tUS treatment.

### EEG recordings and data analysis

The mice were tethered to the recording system and habituated to the recording environment for 24 h before recordings (Fig. 1b). EEG of freely moving mice was recorded with a sampling rate of 2 kHz and a low-pass filter at 100 Hz (8200-K1-SL, Pinnacle Technology, Lawrence, KS) from 00:00 to 08:00. The EEG data were analyzed offline in the customized code with Matlab and Python. Spontaneous gamma power was examined at 30–80 Hz. Briefly, the signal was filtered by a second-order Butterworth filter with a higher and a lower cut-off adjusted to 2 Hz above and 100 Hz below to remove DC noise. Spontaneous gamma power was calculated by computing the power spectral density (PSD) using Welch’s method and integrating the PSD at the given frequency band using the composite Simpson’s rule. The phase locking value (PLV) was calculated by applying the Hilbert transform with a determined frequency band and measuring the differences in the instantaneous phase between the frontal and parietal EEG signals. All analyses were done using a toolbox in MATLAB (Brainstorm, Mathworks). Cross-frequency phase-amplitude coupling (PAC) was calculated by the Gaussian Copula PAC method and denoising.

### Enzyme-linked immunosorbent assay (ELISA)

The mice were deeply anesthetized by isoflurane and then transcardially perfused with phosphate-buffered saline (PBS). Brains were removed and dissected into two hemispheres. The right hemisphere was dissected into the brainstem, thalamus, hippocampus, cerebellum, and cortex and stored at − 80 °C until use. The cortical and hippocampal regions were homogenized in 20 mM Tris–HCl (pH 7.6) with 5 mM EDTA and protease inhibitor cocktail (P3100, GenDEPOT, Katy, TX). The homogenates were centrifuged at 430,000 g for 20 min at 4 °C to separate the soluble and insoluble Aβ. The supernatants were kept at − 20 °C, and the pellet was resuspended in 5 mM guanidine-hydrochloride and 50 mM Tris–HCl (pH 7.6). After centrifugation at 430,000 g for 20 min at 4 °C, the supernatant was collected and stored at -20 °C. The total protein was quantified by BCA assay (23225, Thermo Fisher Scientific, Waltham, MA). ELISA for soluble and insoluble Aβ was conducted following the manufacturer’s instructions (Aβ42: KHB3441, Aβ40: KHB3481, Thermo Fisher Scientific).

### Histology

The left hemisphere was fixed in 4% paraformaldehyde (pH 7.2) in 0.1 M PBS overnight and placed in 30% sucrose in 0.1 M PBS solution until it sank at 4 °C. The fixed brain tissues were cut into coronal sections (40 μm) with a cryostat (Leica Biosystems, Buffalo Grove, IL). The brain sections were rinsed three times with PBS containing 1% Triton X-100 (PBST) for 10 min each after shaking at 120 rpm at room temperature, then they were blocked in 3% normal donkey serum (D9633, Sigma Aldrich, St. Louis, MO) in 0.5% PBST for 2 h at room temperature. The primary antibody used for microglia was rabbit anti-Iba1 (1:1000, 019–19741, Wako, Japan) and the secondary was donkey anti-rabbit IgG (1:500, A31572, Invitrogen, Waltham, MA). Aβ deposits were stained by 1 mM Thioflavin S (T1892, Sigma Aldrich, St Louis, MO). The section was mounted onto a silane-coated slide glass (5116-20F, Muto Pure Chemicals, Tokyo, Japan), and image acquired with a confocal microscope (LSM880NLO, Carl Zeiss, Jena, Germany). Confocal microscopic images were analyzed by the ImageJ program (National Institutes of Health, Bethesda, MD). The mouse brain areas were identified based on Paxinos and Franklin mouse brain atlas [30].

### Perls’ Prussian blue staining

Perl’s Prussian blue staining was performed to detect hemosiderin deposits. We analyzed six brain sections at 2 mm posterior to the bregma, covering retrosplenial area, medial parietal association areas, primary somatosensory area and other cortices and hippocampus. The tissues were rinsed with TBS for 10 min at room temperature. Perl’s Prussian blue staining was conducted following the manufacture’s instructions (ab150674, Abcam, Cambridge, UK). Section was mounted onto a silane-coated slide glass (5116-20F, Muto Pure Chemicals, Tokyo, Japan). Images were acquired by a slide scanner (VS200, Olympus, Tokyo, Japan) and analyzed with the ImageJ program.

### Stereological quantification of Aβ plaques and microglia

Histological images of four serial coronal sections with 200-μm distances in PIL (AP + 2.1, 1.9, 1.7 and 1.5 mm from bregma) and hippocampus (AP + 1.2, 1.4, 1.6 and 1.8 mm from bregma) were obtained with confocal microscopy (FV3000RS, Olympus, Tokyo, Japan). Aβ plaques and microglia were quantified using an unbiased stereological dissector method by ImageJ software. The images of regions of interest were acquired using a 40 × objective according to the atlas of Paxinos and Franklin [30]. The average thickness of mounted sections was approximately 38 μm. The optical dissector height was 10 μm, with 1-μm distances between consecutive images. In each region of interest, Aβ plaques and microglia were counted in a frame size of 120 μm × 120 μm (PIL) or 200 μm × 200 μm (hippocampus), and a grid size of 250 μm × 250 μm (PIL) or 420 μm × 420 μm (hippocampus).

### Analysis of microglial morphology

Z-stacked images of Iba1 immunohistochemistry were projected with maximum intensity and converted to binary and skeletonized images using ImageJ. The number of microglia was manually counted for each image. The Analyze Skeleton Plugin was used to measure microglial ramification. For each section, the total number of endpoints and the length of microglial processes were normalized to the number of microglia.

### Data analysis

Statistical analyses for histological data were performed using the Prism 9 software (GraphPad Software, Inc., La Jolla, CA), and electrophysiological data were analyzed by RStudio software (RStudio, Boston, MA). Independent *t*-test or Mann–Whitney Rank Sum tests were used to compare two groups; one-way ANOVA with Tukey’s *post-hoc* test was used to compare three groups. Fisher’s exact test was used in the analysis of contingency table.

## Results

### No increase in intracerebral microbleeds after tUS stimulation

**Fig. 2:**
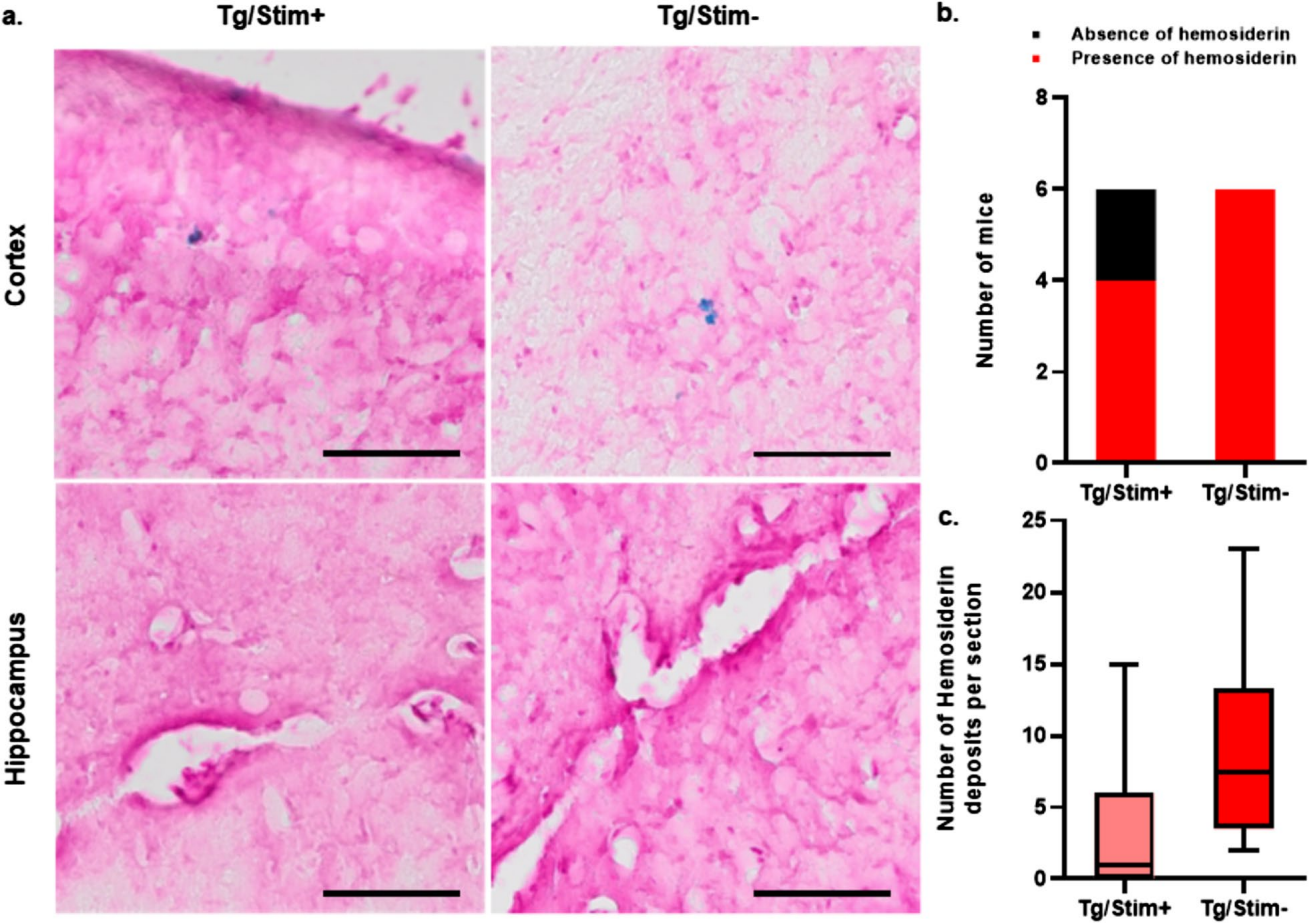
Perls’ Prussian blue staining to detect mouse brain damage. (**a**) Representative images of Perl’s Prussian blue staining in each mouse group. Blue pigments in the cortex indicate hemosiderins. No hemosiderin-positive deposit was found in the hippocampus. Scale bar, 100 μm. (**b**) Numbers of mice with or without hemosiderin deposits (*P* = 0.455 by Fisher’s exact test). (**c**) Numbers of hemosiderin deposits per section compared between the treatment groups. Box plots show medians and confidential intervals of 95% (*P* = 0.064 by Mann–Whitney U test; *n* = 6 for each group)

To examine the potential toxic effects of the stimulation, we quantified the hemosiderin deposits using Perls’ Prussian blue staining to compare the intracerebral microbleeds (Fig. 2a). The numbers of mice with hemosiderin deposits did not differ significantly between the tUS and sham stimulation groups (*P* = 0.455 by Fisher’s exact test; Fig. 2b). Instead, the median number of hemosiderin deposits per brain section was lower in the Tg/Stim+ group, although there was no statistical significance (Fig. 2c).

### Aβ loads in cortical and hippocampal regions changed after two weeks of tUS at 40 Hz

**Fig. 3:**
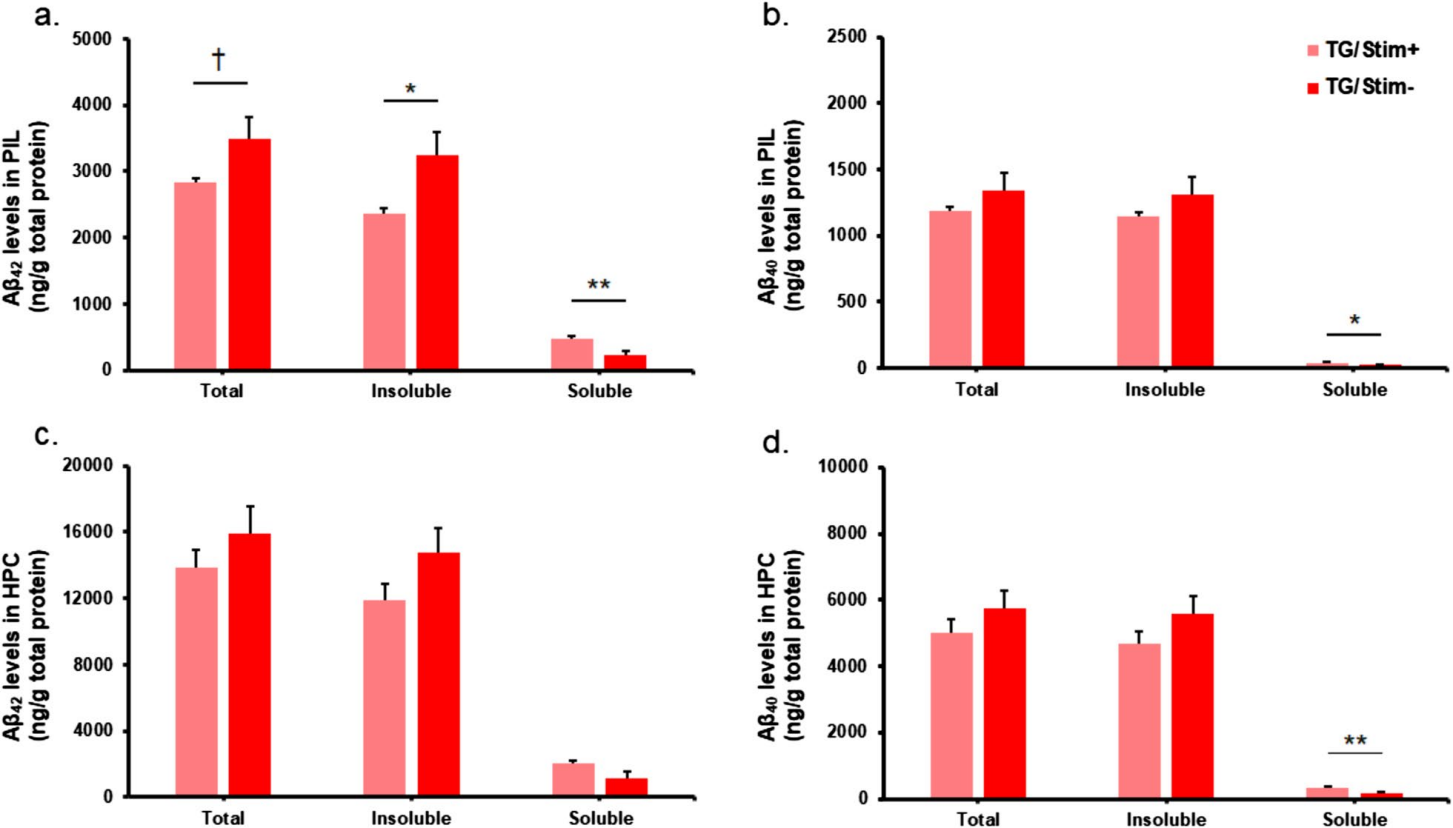
ELISA analysis of Aβ load in PIL and the hippocampus. **a, b** Total, insoluble and soluble Aβ42 and Aβ40 levels in PIL. **c, d** Total, insoluble and soluble Aβ42 and Aβ40 levels in the hippocampus. Pink and red bars indicate 5×FAD mice with stimulation (Tg/Stim+; *n* = 6), and 5×FAD mice with sham stimulation (Tg/Stim-; *n* = 6), respectively. PIL, pre- and infra-limbic cortex; HPC, hippocampus; ^†^*P* < 0.1, *P < 0.05, **P < 0.01, independent *t*-test or Mann–Whitney Rank Sum test

**Fig. 4:**
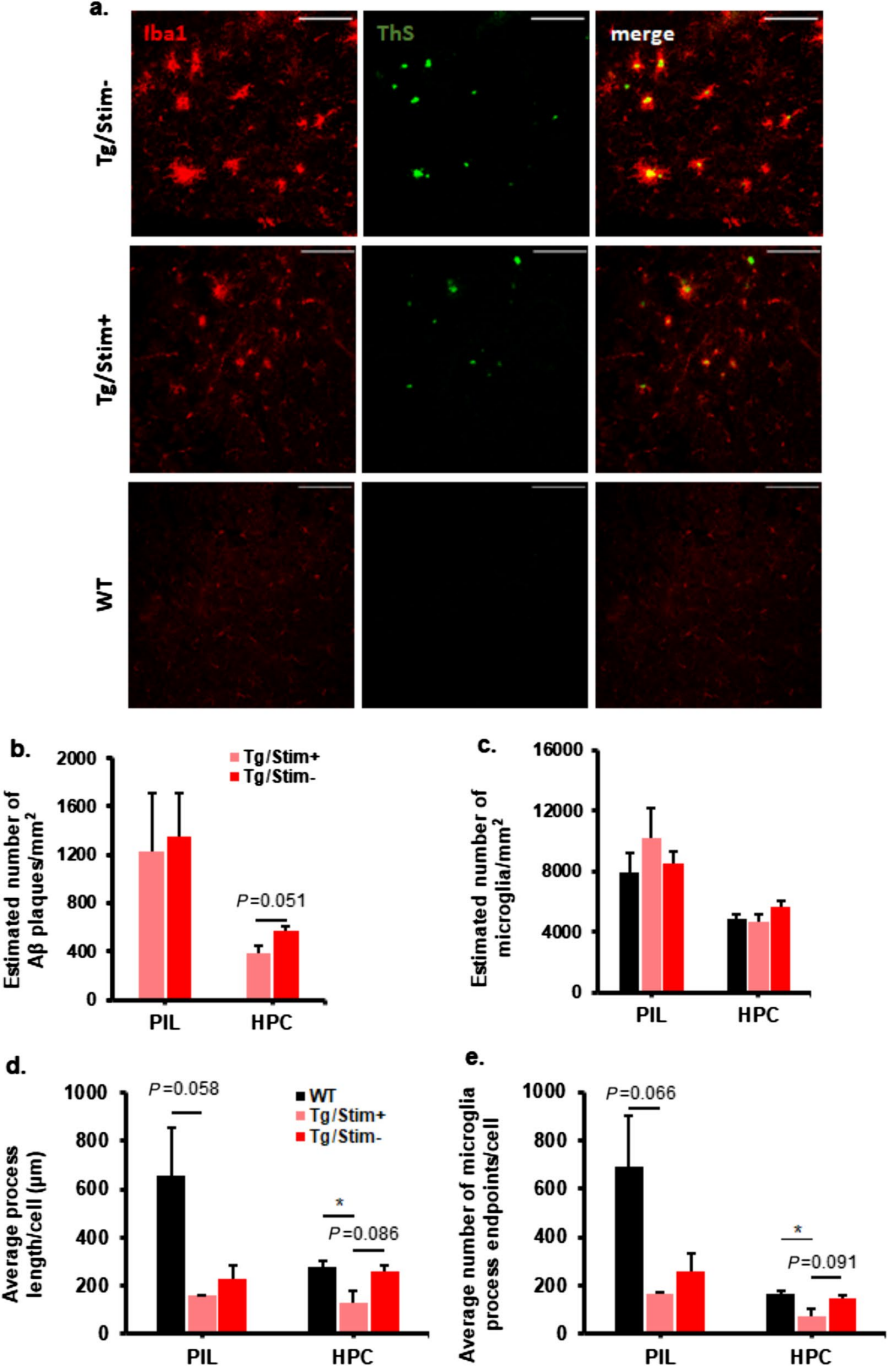
Quantification of Aβ plaques and microglia and morphological analysis of microglia. **a** Representative images of microglia and Aβ plaque in PIL (Scale bar, 100 μm). **b** Estimated number of Aβ plaques per unit area in PIL and HPC (*P* = 0.051 by two-sample *t*-test) analyzed by unbiased stereology. **c** Estimated number of microglia per unit area in PIL and HPC analyzed by unbiased stereology. **d** Average length of microglial processes per cell in PIL and HPC. **e** Average number of microglia process endpoints per cell in PIL and HPC. *P < 0.05, one-way ANOVA with Tukey *post-hoc* test, *n* = 3

In the PIL, there was a significant decrease in total Aβ42 in the Tg/Stim+ group compared to the Tg/Stim- group (Fig. 3a). Sub-fraction analysis showed a significant reduction of insoluble Aβ42 and increase of soluble Aβ42 levels in the Tg/Stim+ group compared with the Tg/Stim- group (*P* < 0.05; Fig. 3a). We also observed a decrease in total and insoluble Aβ40 levels in the Tg/Stim+ group compared with the Tg/Stim- group, but the difference was not significant (Fig. 3b). In the hippocampus, Aβ level showed similar patterns of change, but the difference was not significant (*P* > 0.05; Fig. 3c, d). Unbiased stereological analysis showed that in the hippocampus, the estimated number of Aβ plaques per unit area was diminished in the Tg/Stim+ group compared to the Tg/Stim- group (*P* = 0.051; Fig. 4a, b). The Tg/Stim+ did not show significant differences in the estimated number of microglia per unit area from the Tg/Stim- group in PIL and hippocampus (Fig. 4c). To examine microglial activation, we analyzed the morphology of microglia in PIL and hippocampus. Microglia in the Tg/Stim+ group showed a reduced number of process endpoints and decreased process length in the PIL compared to WT (Fig. 4d, e). Also, the hippocampal microglia of the Tg/Stim+ group showed a trend of decrease in the length and the number of processes compared to the Tg/Stim- group (*P* = 0.086 and 0.091, respectively; Fig. 4d, e).

### Relative spontaneous gamma power and phase-locking value in EEG changed after 2 weeks of tUS at 40 Hz

**Fig. 5:**
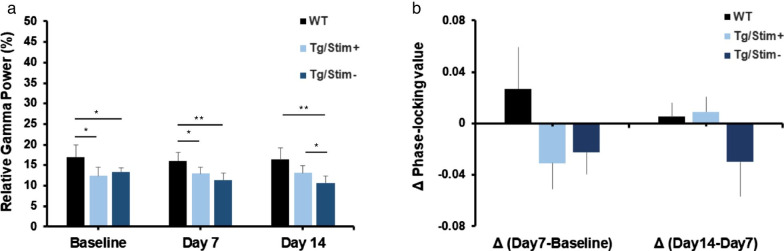
Spontaneous gamma power and phase-locking value increased after two weeks of ultrasound stimulation. **a** EEG gamma power relative to total power. **b** Change in phase-locking value from baseline to stimulation on day 14. WT, wild type; Tg/Stim+, 5×FAD with stimulation; Tg/Stim-, 5×FAD with sham stimulation; *P < 0.05, **P < 0.01, one-way ANOVA with Tukey *post-hoc* test

Power spectral analysis (Welch’s method) was performed with a range of gamma frequencies (30–80 Hz). The gamma power was then normalized to the total power. ANOVA and *post-hoc* multiple comparisons revealed that at baseline and after 7-day stimulation, both Tg/Stim+ and Tg/Stim- groups had significantly lower spontaneous gamma power than the WT mice (Fig. 5a). However, after 14-day stimulation, the spontaneous gamma power of 5×FAD mice with tUS stimulation was significantly higher than that of 5×FAD mice without tUS stimulation (*P* < 0.05), and became comparable to that of the WT mice (*P* > 0.05).

PLV was calculated at the spontaneous gamma frequency, and differences of PLV at day 7 *versus* baseline and at day 14 *versus* day 7 were calculated for each group, to investigate the change of synchronization of spontaneous gamma between frontal and parietal EEG signals. There was an increase of PLV from baseline to day 7 in the WT group, but both Tg/Stim+ and Tg/Stim- groups showed a downward trend (Fig. 5b). In addition, from day 7 to day 14, the PLVs in Tg/Stim+ and WT groups showed a trend of increase, whereas the PLV of the Tg/Stim- group remained reduced (Fig. 5b).

### Cross-frequency PAC changed after 2 weeks of tUS stimulation at 40 Hz

**Fig. 6:**
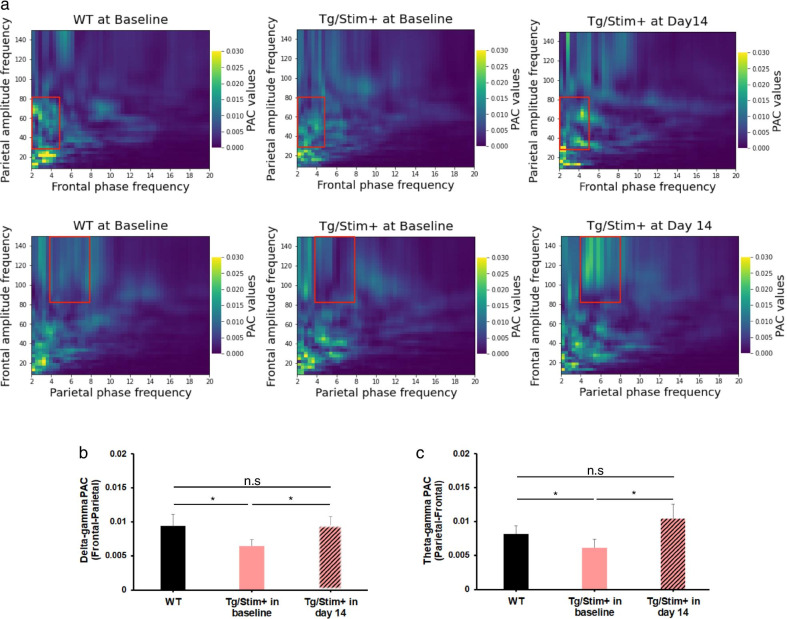
Cross-frequency phase-amplitude coupling was improved after 2 weeks of ultrasound stimulation. **a** Cross-frequency phase-amplitude coupling (PAC) of the frontal and parietal EEG signals. Upper: parietal amplitude frequency and frontal phase frequency coupling; bottom: frontal amplitude frequency and parietal phase frequency coupling. Red rectangles represent the area of interest. **b, c** Delta-gamma and theta-gamma PAC. WT, wild type; Tg/Stim+, 5×FAD with stimulation; n.s., no significance; *P < 0.05, **P < 0.01 following two-sample (independent) *t*-test

Regarding PAC, a decreased coupling of delta-phase (2–5 Hz; frontal EEG) and gamma-amplitude (30–80 Hz; parietal EEG) at baseline in Tg/Stim+ was observed compared to WT, but this coupling was improved on the last day of 14-day stimulation (Fig. 6a upper panels and 6b). The coupling of theta-phase (5–8 Hz; parietal EEG) and gamma-amplitude (80–160 Hz; frontal EEG) increased in Tg/Stim+ after 14-day stimulation compared to that at baseline (Fig. 6a lower panels and 6c). Due to the heterogeneous types of data in the three groups, we performed pairwise comparisons using the independent and paired *t*-test.

## Discussion

To suggest tUS as a treatment modality, it is of utmost importance to exclude potential toxicity. In AD, cerebral amyloid angiopathy is three times more prevalent than in the general population [31]. In this study, we found no increase in microbleeds after tUS stimulation compared to the sham group, suggesting that tUS is a safe neuromodulatory tool with the present stimulation protocol. Indeed, the energy density of our tUS stimulation was under the safety limit. Moreover, here we observed that the 14-day ultrasonic stimulation at 40 Hz gamma frequency decreased Aβ load in PIL and hippocampus in an animal model of AD. In addition to the molecular and histologic improvement, we found neurophysiological evidence of functional improvements, such as increased spontaneous gamma oscillations, PLV, and PAC throughout the treatment course. The novelty of our approach is to apply non-focusing ultrasound pulsed at 40 Hz, which may be an optimal stimulation modality to achieve efficacy and safety. However, the stimulation protocol must be optimized to achieve improved efficacy and safety by systematic trials with more extended stimulation periods, higher energies, and other frequencies in future studies.

### Neuromodulatory effects of ultrasound stimulation

Recent studies have already shown that ultrasound stimulation can exert neuroprotective effects. Eguchi et al. [25] demonstrated that ultrasound stimulation with a pulse repetition frequency of 1 kHz could reduce Aβ plaque, increase cerebral blood flow, and affect endothelial nitric oxide that is considered as an important therapeutic target for AD [5]. Additionally, Huang et al. have reported that low-intensity pulsed ultrasound is effective in improving the density of dendritic spines, altering electrophysiological properties, and increasing the expression of *N*-methyl-*D*-aspartate (NMDA) receptor 2A in the hippocampus [26]. All of these findings provide strong evidence for the beneficial effects of ultrasonic neuromodulation. Here, ultrasound stimulation with pulse repetition frequency of ~ 300 kHz was selected due to its higher efficacy in human and animal brain neuromodulation compared with other higher frequencies. Previous studies [32, 33] have shown that neuromodulation in vivo is more effective at frequencies less than 1 MHz, especially 300 kHz, than at 1 MHz. In addition, here the tUS stimulation pulsed at 40 Hz resulted in a strong response, similar to the auditory steady-state response to the click sounds at 40 Hz [34]. Although we focused on the impacts of gamma oscillations, effects from other frequencies, which are beyond the scope of the current study, cannot be excluded.

### Advantage and disadvantage of gamma entrainment by tUS

Gamma entrainments of neurons have been suggested as one of the promising methodologies for AD treatment. Iaccarino et al. showed that neuronal entrainment at gamma frequency by light flickering or optogenetics could reduce Aβ in an animal model with Aβ overproduction, by enhancing the phagocytic activity of microglia in the brain [23]. Subsequently, the same group showed that visual and auditory entrainment at 40 Hz could also improve Aβ pathology and cognitive function [24, 35]. These gamma entrainments via sensory neural systems, such as visual and auditory pathways, have advantage of safety and thorough engagement of the implicated neural networks due to the usage of the endogenous systems, but their driving efficiency can be limited by the individuals’ neural system *per se*. For example, if the sensory systems are affected by the pathological process, the effect of gamma entrainment will reduce accordingly. It is noteworthy that hearing and visual disturbances are not uncommon in the elderly. On the other hand, focused ultrasound stimulation may increase the risk of inflammatory response and tissue damage, such as intracerebral microbleeds. Therefore, a novel methodology that can enable gamma entrainment of the brain in a non-invasive and wide-ranging manner is required. We suggest that the tUS stimulation can address these problems in AD treatment with gamma entrainment.

### tUS stimulation can entrain gamma band oscillations

Fast-spiking parvalbumin (PV) cells are GABAergic interneurons that account for ~ 40% of inhibitory interneurons and receive NMDA excitatory input from pyramidal cells [17, 36]. The regulation of fast-spiking PV interneurons through GABAergic inhibitory synaptic activity onto excitatory pyramid cells generates and fine-tunes gamma oscillations [13–15]. Gamma oscillations in neural networks play an important role in perception and cognition, including the allocation of attention and working memory. Previous studies have shown that the gamma oscillation is reduced in AD [17, 21]. In 2012, Verrett et al. reported recovery of the gamma activity by increasing Nav1.1 expression in PV cells followed by the reduction of network hyper-synchronization and cognitive function loss in the AD animal model [17]. In our study, we observed that tUS at 40 Hz could entrain the gamma-band oscillations. The relative gamma power increased significantly after 2 weeks of ultrasound stimulation, whereas that of the non-treatment group (Tg/Stim-) remained significantly decreased compared to WT.

We speculate that the improvement of spontaneous gamma may be related to the reduction of insoluble Aβ. Indeed, Aβ can trigger Th1 cells to release IFN-γ [37], resulting in disturbed neuronal network oscillations via microglial activation and increased nitric oxide [38]. Furthermore, we observed morphological changes of microglia that imply microglial activation. Recently, it has been reported that gamma entrainment by optogenetics or visual stimulation enhances the phagocytic activity of microglia [23]. Depending on the polarization, microglia can be either pathogenic or therapeutic. The activated microglia in our experiment might also signify a microglial phenotype switch from pro-inflammatory M1 to anti-inflammatory M2 subtype. Unfortunately, our histological marker Iba1 could not distinguish the subtypes of microglia. Considering that the M2 microglia have increased phagocytic activity, we speculate that tUS may reduce insoluble Aβ by phagocytic activity of M2 microglia. In addition, since GABA tonically released from reactive astrocytes might play a role in memory dysfunction [39] and, potentially, impaired gamma oscillations, we also postulate that the decreased neuroinflammation could relieve the reactive astrogliosis and improve the gamma oscillations.

### Aβ load decreased by tUS

The total Aβ levels (Aβ42 and Aβ40) in cortical areas and the hippocampus were reduced after two weeks of tUS, compared to the Tg/Stim- group. The decrease in insoluble Aβ42 in the Tg/Stim+ group was significant in the cortex but not in the hippocampus, whereas soluble Aβ42 increased slightly but significantly compared to the Tg/Stim- group. Thus, in terms of Aβ level, tUS showed both therapeutic and potentially harmful effects simultaneously. The relative toxicity of soluble and insoluble Aβ is controversial. Insoluble Aβ fibrils aggregate from Aβ monomers and oligomers [40], and they can accumulate to form amyloid plaques, known to be pathological hallmarks in AD. The aggregation of insoluble fibrils causes neuroinflammation, which leads to neurite damage and decreases Aβ clearance [41, 42]. The fibrillar plaques are also causally related to progressive neuritic abnormalities in amyloid precursor protein transgenic mice [43, 44]. Indeed, our histological analysis showed reduced number of Aβ plaques in the hippocampus and decreased length and endpoints of microglial processes in the Tg/Stim+ group, implying the activation of microglia [23, 45].

According to our computational simulation of the affected regions by tUS stimulation, more than 60% of ultrasound wave pressure was transmitted through the mouse skull and reached the brain (Additional file 1: Fig. S1). Depending on the threshold value (− 6 dB *vs* − 12 dB) of the maximum simulatory acoustic pressure in the brain, 9% or 45% of brain volume was affected by tUS stimulation, respectively, even though the minimum acoustic pressure to stimulate the brain remains unclear. These results proved that the unfocused tUS affects a significant portion of the brain compared to focused tUS. Particularly, the region near the hippocampus appears to receive a stronger intensity of the ultrasound beams than PIL, resulting in more robust effects in histology.

The decrease in insoluble Aβ detected in this study may result in reduced neuroinflammation, or vice versa, in spite of the increase in soluble Aβ. However, amyloid oligomers are also soluble and can spread widely throughout the brain. The soluble oligomers can cause hyper-phosphorylation of tau protein, which forms neurofibrillary tangles and leads to neuronal synaptic dysfunction in AD [46]. Aβ oligomers also induce the disruption of the neuritic cytoskeleton and accelerate cytotoxic effects [47]. Both fibrils and soluble Aβ have been found to induce cell death via different pathways: oligomers induce cell death via apoptosis, whereas amyloid fibrils lead to necrosis-like death [48]. Given that both insoluble and soluble Aβ are pathogenic via the sum of various mechanisms, such as neuroinflammation, synaptotoxicity and tau pathology, we may be able to suggest that the net effect of the tUS gamma entrainment might be therapeutic in spite of increased soluble fraction of Aβ. On the other hand, the timing of effects on Aβ may be different from previous research. The tUS stimulation might have a later therapeutic effect on the functionality of the brain than auditory or visual stimulation [23, 24], as in our consecutive analysis, the spontaneous gamma power showed significant improvement on day 14, but not on day 7. Direct comparison with the previous report on day 7 was not possible because we sampled the brain only on day 14. Nevertheless, since there is controversy on Aβ reduction as a therapeutic strategy, as was discussed recently on aducanumab [49], the importance of functional improvement may be more crucial as a direct indicator of therapeutic effect.

### Functional improvement could be achieved by tUS

Recent studies have reported that the loss of EEG synchronization increases in AD patients and is correlated with cognitive dysfunction [20–22]. The gamma-band entrainment technique can improve cognitive function and memory performance [23, 24]. Because tUS at 40 Hz can increase the spontaneous gamma power, we investigated whether tUS could provide functional improvement in the 5×FAD mouse model. PLV can effectively reflect functional impairment of connectivity because it measures the temporal relationships of neural signals independent of their amplitude. Our results showed a decreasing trend in PLV in the sham treatment group (Tg/Stim-) over two weeks, while the direction of PLV changed in the treatment group (Tg/Stim+) from decrement (day 7 − baseline) to increment (day 14 − day 7), although there was no statistical significance. This suggests that the two weeks of ultrasound stimulation might have contributed to the recovery of PLV. We also analyzed the cross-frequency PAC to see how the phase of low-frequency oscillations modulates high-frequency power, especially the gamma band. The local processing could be fine-tuned or influenced by the modulation of gamma activity within particular areas by low-frequency oscillation. Recent studies revealed that the theta-phase high-gamma coupling correlates with working memory by manipulating the ordering of information during the working memory process [39, 40]. Impairment of theta-gamma coupling followed by memory deficits has been reported by Goodman et al. in 2018 [41]. In our study, we observed reduction of theta-gamma PAC in Tg/Stim+ mice compared with WT at baseline. After 2 weeks of tUS stimulation, the theta-gamma coupling in Tg/Stim+ mice increased significantly in comparison to the baseline. Delta-gamma coupling is also considered as a biomarker for evaluating generalized EEG suppression and network activity [42]. Our results showed increased delta-gamma coupling in Tg/Stim+ mice on day 14 compared to baseline. Our results are consistent with a previous study by Yi Yuan et al., which showed that the PAC indices between the delta, theta, alpha, and gamma oscillations increased significantly with ultrasound stimulation [50], and thereby concluded that the ultrasound stimulation could be a powerful noninvasive method to interfere with the brain by modulating gamma oscillations in the rat hippocampus. Taken together, all of these results support that gamma-band entrainment by tUS stimulation at 40 Hz can normalize PLV and cross-frequency coupling, implying improved brain connectivity and information processing.

### Ultrasound stimulation in AD treatment

Recently, several clinical studies have reported that ultrasound stimulation is a potential methodology to improve brain function and even cortical thickness in human AD patients. The promising results were replicated in independent groups over the last years, showing that transcranial pulse stimulation with focused ultrasound could improve neuropsychological scores and increase cortical thickness [51, 52]. In addition, low-intensity transcranial focused ultrasound (LI tFUS) has been reported with potential effects in AD treatment by Jeong et al. [53]. In their study, LI tFUS targeting the hippocampus has a beneficial impact on cerebral glucose metabolism and memory and improves cognitive function. Thus, a large body of preclinical and clinical evidence suggests that the blood–brain barrier (BBB) opening by FUS can be done safely [54, 55]. However, potential risks should be noted, including microbleeds, cellular vacuolation, ischemia due to vasoconstriction, cerebral edema, and direct cellular injury from physical forces, even at the typical low sound pressure [56]. Since most neuromodulation studies have been done without BBB opening and generated clear neurophysiologial effects, non-BBB opening ultrasound ("focused" and "unfocused") might be a safer therapeutic option.

## Conclusion

In this study, two-week tUS decreases total Aβ42 and Aβ40 levels, especially insoluble Aβ, in the brain cortex and hippocampus of 5×FAD mice. The reduction of Aβ plaque is comparable to the recent visual and auditory stimulation studies in AD mouse models. In addition, tUS stimulation increases spontaneous gamma power and PAC, indicative of functional improvement. In summary, tUS brain stimulation at 40 Hz can be a potential therapeutic modality by reducing Aβ load and improving brain connectivity. In future studies, the exact neurobiological mechanisms of these effects need to be investigated.

## Supplementary Information

**Additional file 1**: **Fig. S1.** Simulated acoustic pressure distribution in the brain during transcranial ultrasound simulation.

## Data Availability

The datasets used and/or analysed in the current study are available from the corresponding authors on reasonable request.

## Abbreviations

AD: Alzheimer’s disease
Aβ: Amyloid-β
EEG: Electroencephalogram
WT: Wild type
tUS: Transcranial ultrasound stimulation
PSD: Power spectral density
PLV: Phase locking value
PAC: Phase-amplitude coupling
ELISA: Enzyme-linked immunosorbent assay
PBS: Phosphate-buffered saline
PIL: Pre- and infra limbic
RM ANOVA: Repeated measure analysis of variance
PV: Parvalbumin
FS-PV: Fast-spiking parvalbumin
NMDA: *N*-Methyl-*D*-aspartate
