# Supplementary material for: Effects of transcranial ultrasound stimulation pulsed at 40 Hz on Aβ plaques and brain rhythms in 5×FAD mice
Source: Transl Neurodegener. 2021 Dec 7;10:48. doi: 10.1186/s40035-021-00274-x (PMC8650290; doi:10.1186/s40035-021-00274-x)
Supplement: Supplementary file 1 — Additional file 1: Fig. S1. Simulated acoustic pressure distribution in the brain during transcranial ultrasound simulation. [file 40035_2021_274_MOESM1_ESM.docx]

**Supplementary Materials**

**Simulation of the acoustic pressure distribution by transcranial unltrasound stimulation**

The 3D transcranial ultrasound simulation was performed using a k-Wave toolbox of MATLAB in order to examine the acoustic pressure distribution over the brain [1]. The mesh of the cranium after extracting cranium structures from the CT images was imported into MATLAB to form a voxel for simulation, followed by filling the water inside the cranium as shown previously [2-5]. For the simulation, the sound speed of the water was 1500 m/s, and that of a mouse cranium was 2425 m/s. The density of water was 1000 kg/m3, while 1933 kg/m3 was used as the density value of the cranium. The attenuation coefficients of water and the cranium were 0.0022 and 5.62 (dB/cm/MHz), respectively [3]. In terms of the spatial and temporal step sizes, 62.5μm (𝜆/80) and 7.73 ns were used in the simulation space. Since the minimum pulse duration was 3ms, the simulation was conducted to see the acoustic pressure pattern during and after the ultrasound stimulation for 5ms.


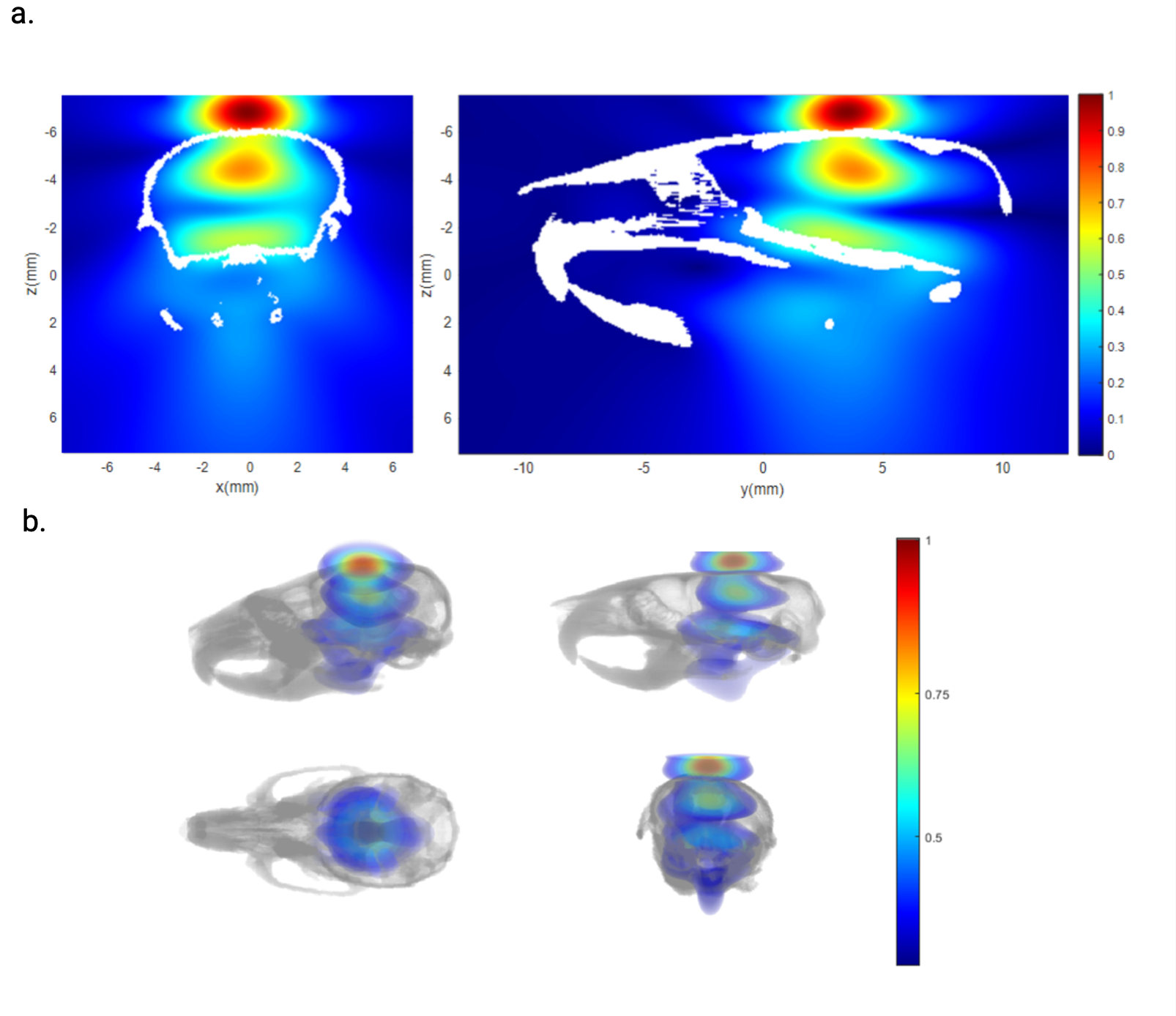


**Fig. S1. Simulated acoustic pressure distribution in the brain during transcranial ultrasound simulation.** (**a**) Sagittal and coronal distribution of acoustic pressure in the brain when tUS was applied to the skull 1.5 mm above. (**b**) 3D results of simulated acoustic pressure distribution in the brain from tUS simulation. The volume fraction of the brain stimulated by the acoustic beams was 9% and 45%, respectively, when the threshold was -6dB and -12dB of the maximum acoustic pressure value.

**References:**

1. Treeby BE, Cox BT. k-Wave: MATLAB toolbox for the simulation and reconstruction of photoacoustic wave fields. J Biomed Opt. 2010;15:021314.

2. Liang B, Liu W, Zhan Q, Li M, Zhuang M, Liu QH, et al. Impacts of the murine skull on high-frequency transcranial photoacoustic brain imaging. J Biophotonics. 2019;12:e201800466.

3. Kim S, Jo Y, Kook G, Pasquinelli C, Kim H, Kim K, et al. Transcranial focused ultrasound stimulation with high spatial resolution. Brain Stimulation. 2021;14:290–300.

4. Mueller JK, Ai L, Bansal P, Legon W. Numerical evaluation of the skull for human neuromodulation with transcranial focused ultrasound. J Neural Eng. 2017;14:066012.

5. Wu SY, Aurup C, Sanchez CS, Grondin J, Zheng W, Kamimura H, et al. Efficient blood-brain barrier opening in primates with neuronavigation-guided ultrasound and real-time acoustic mapping. Sci Rep. 2018;8:7978.
